# “Work WITH us”: a Delphi study about improving eating disorder treatment for autistic women with anorexia nervosa

**DOI:** 10.1186/s40337-023-00740-z

**Published:** 2023-02-09

**Authors:** Sarah L. Field, John R. E. Fox, Catherine R. G. Jones, Marc O. Williams

**Affiliations:** 1 South Wales Doctoral Programme in Clinical Psychology, Cardiff University, 11Th Floor, Tower Building, 70 Park Place, Cardiff, CF10 3AT UK; 2 Doctorate in Clinical Psychology, University of Liverpool, Liverpool, L69 7ZA UK; 3 Wales Autism Research Centre, School of Psychology, Cardiff University, Cardiff, CF10 3AT UK

**Keywords:** Autism, Eating disorder, Anorexia nervosa, Delphi study

## Abstract

**Background:**

There is an increased prevalence of anorexia nervosa (AN) in autistic women and this group has poorer treatment outcomes compared to non-autistic women with AN. However, there is little research into improving eating disorder treatment for autistic women. This study investigated how best to support autistic women with AN within eating disorder services.

**Method:**

A three-stage Delphi study was conducted with 49 participants with relevant expertise as a researcher, clinician, or expert by experience.

**Results:**

A total of 70 statements were generated, with 56 reaching consensus after the final round. Statements reaching consensus made recommendations for adaptations to treatment, staff training, and service organisation.

**Conclusions:**

The results highlight the need to distinguish between autism- and AN-related difficulties, accommodate autistic traits such as sensory sensitivities and communication differences, and ensure the autistic voice is present in both the development and delivery of care. Future research should investigate the impact of these adaptations on outcomes. The applicability of these recommendations to autistic people with other eating disorders and of other genders needs to be investigated further.

**Supplementary Information:**

The online version contains supplementary material available at 10.1186/s40337-023-00740-z.

## Plain English summary

Autistic women are more likely to have anorexia nervosa (AN) than non-autistic women. Autistic women can find eating disorder treatment unhelpful and need adaptations to treatment. This study asked a group of 49 researchers, staff, and people with personal experience of autism and eating disorders what they thought would help autistic women with AN. The study used a Delphi study method, which allows the calculation of how much participants agree without them needing to meet and make a decision. The study created 56 suggestions that the participants agreed on. The results give suggestions for changing treatment, training staff, and changing how services work to be better for autistic women. The suggestions highlight the importance of being able to tell the difference between autism- and AN- related behaviour, adjusting care to accommodate autistic traits, and involving autistic people in the development of care. Many of the suggestions recommend that changes are flexible to the individual autistic person. In the future, research should check if these changes are helpful for autistic women with AN, and if they would be helpful for autistic people who are not female or have other eating disorders.

**Supplementary Information:**

The online version contains supplementary material available at 10.1186/s40337-023-00740-z.

## Background

Autism is a neurodevelopmental condition characterised by difficulties with social communication and interaction, as well as the presence of restricted or repetitive interests or behaviours [1]. Co-occurring mental health difficulties are common in autistic people, with prevalence rates exceeding those found in general population samples [2]. When autistic people and other stakeholders were consulted, one of their top priorities was research into the mental health of autistic people [3].

Anorexia nervosa (AN) is an eating disorder (ED) characterised by restricted food intake and an intense fear of or behaviour designed to prevent gaining weight [1]. AN has a significant impact on individuals’ functioning and quality of life, with high levels of mortality [4], and is particularly prevalent among females [5]. Autistic women are overrepresented amongst people receiving treatment for AN [6, 7]. On ED inpatient admission, adults with higher Autism Quotient (AQ-10; 8) scores had higher levels of ED psychopathology [9]. People with AN and higher levels of autistic traits also had longer illness durations [10]. Autistic women with AN reported accessing a broader range of ED treatments than non-autistic women, suggesting they have difficulty accessing treatments that work [11]. Autistic women also rated various aspects of ED treatment as less beneficial than non-autistic women [11]. When followed up over a 30 year period, autistic adolescents with AN had worse outcomes than non-autistic adolescents in terms of mental state, independence from their families, social contact, and employment [12].

Brede et al. [13] autism-specific model of restrictive eating difficulties suggests autism-related traits can both directly and indirectly lead to restricted eating. For example, special interests related to food and exercise may be a direct path to restricted eating. Other traits may indirectly lead to restricted eating as a way to cope with negative emotional experiences. For example, emotional distress caused by social difficulties may be numbed through restricting food intake [13]. Perhaps reflecting this complexity, clinicians reported lacking confidence working with autistic people with AN, and identified difficulties differentiating between autistic characteristics and symptoms of AN [14]. Autistic women described being refused treatment by ED services and being viewed as uncooperative because of their difficulties with traditional treatments [15, 16].

Despite evidence of poor outcomes and treatment experiences for autistic women with AN, there is little research into how treatment can be improved for this population. Currently, there is only one set of guidelines on treatment for autistic people with AN [17]; The Pathway for Eating Disorders and Autism developed from Clinical Experience (PEACE) pathway [18, 19]. The PEACE pathway is a practice-based guideline developed as a quality improvement project involving patients, clinicians, carers and researchers within South London and Maudsley NHS Trust Eating Disorder Services. Recommendations from the pathway include training staff in the interaction between autism and EDs, adaptation of the ward environment, and the provision of a specialist menu [19]. As the PEACE pathway has only recently been developed, there is limited research into its impact. However, the length of inpatient admission for autistic people with AN reduced after the pathway was developed [20].

Due to the nature of quality improvement projects, there is limited detail on how the PEACE pathway was developed. It is unclear to what extent different stakeholders contributed to the development of the guidelines, the diversity of stakeholder’s experiences, and at which stages input was provided. As the project was developed within a specific service, it is unclear whether the recommendations would be appropriate for other services. Systematic research is required to further explore how treatment for autistic women can be improved.

The present study used a Delphi method to develop a consensus on how to support autistic women during treatment for AN. This approach is recommended when there is not an accepted body of knowledge on a topic [21]. Delphi studies emulate group decision making in a controlled context to reduce the impact of biases [22]. An advantage of this is experts from different backgrounds have an equally weighted impact on the results. The Delphi method enables a transparent view of the decision-making process and how strongly participants agree with the results.

Delphi studies vary methodologically; they typically involve participants rating their agreement with a set of statements generated from a literature search [23, 24] or qualitative responses from a group of experts [25, 26]. A facilitator provides participants with feedback on how their ratings compare to the group and allows them to revise their responses. Over several rounds of questionnaires, responses converge and a statistical criterion is used to define which statements meet consensus [27].

The quality of Delphi studies’ results depends on the expertise of participants [28]. Groups with diverse expertise make better decisions, providing a rationale for recruiting experts from a variety of professional backgrounds [27]. There is a growing emphasis on increasing the participation of autistic people in research [29, 30], which also improves the relevancy and quality of findings [31]. As a result, participants in the present study were from diverse backgrounds in the areas of autism and EDs, as researchers, clinical staff members, or experts by lived experience (EbE). Our aim was to use a transparent and systematic research method to identify suggestions for improving ED treatment for autistic women.

## Method

### Participants

Participants were recruited via adverts on social media and the researchers’ personal contacts. Participants self-identified as experts in the areas of autism, EDs, or both. They reported whether their expertise was via academic research, clinical work, or personal experience as a carer, autistic person and/or person with an ED (EbE). Participants with multiple sources of expertise were instructed to select the area in which they felt most expert. For EbE, lived experience of either condition or experience as a carer was the primary criterion for inclusion in the study. We purposefully recruited EbE with additional experience of advocacy or service development to enrich the data, however EbE were not required to have this experience in order to participate.

We aimed to recruit 10–18 experts to each of three expertise ‘panels’ [28]: researchers, clinicians, and EbE. Demographic information was gathered to ensure potential experts were suitably qualified, including the number of years of research, clinical, or service development/advocacy experience each expert had. Participants were asked which country they lived or worked in, with an aim to recruit an international sample. Demographic details of the participants are given in the results section. Participants gave informed consent and the research was approved by the University School of Psychology Ethics Committee.

**Fig. 1:**
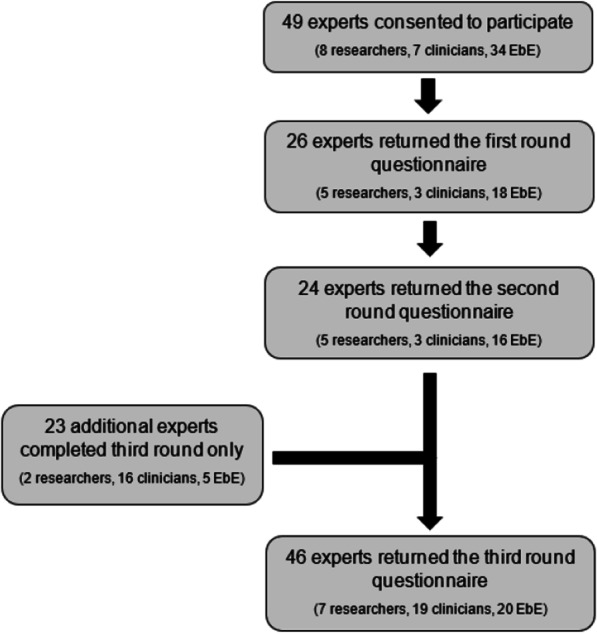
A flowchart showing the number of experts participating at each stage of the Delphi study. *Note*: EbE = expert by experience; ED = eating disorder. Brackets indicate the number of experts in each panel

Figure 1 shows the number of experts participating at each stage of the Delphi study. Due to attrition, additional participants were recruited during the third round. Previous Delphi studies used separate samples for different stages of the study [24] with some combining ratings across stages when calculating consensus [32]. In total, 49 participants completed at least one stage of the study.

### Materials and procedure

The present study used a three-stage Delphi method based on the steps described by Heather et al. [25] and Tierney and Fox [32]. At each stage participants received a survey to complete at their convenience over a couple of weeks.

During round one, participants were emailed an editable document containing six open-ended questions (see Table 1). The questions were selected to elicit answers that could provide insights into improving support for autistic women with AN within ED services.

**Table 1:** Open-ended questions sent to experts in round one

	Question
1	What do you see as the specific needs of autistic women with anorexia nervosa?
2	How are these different to neurotypical women with anorexia nervosa?
3	What do you think would help an autistic woman recover from anorexia nervosa?
4	What is the best way for staff to support an autistic woman with anorexia nervosa?
5	What are the challenges for staff to work with an autistic woman with anorexia nervosa?
6	What would a training package for staff working with autistic women with anorexia nervosa need to achieve?

During the second round, participants were emailed a link to an online questionnaire with the statements generated from content analysis of the results of the first questionnaire. Participants were asked to rate their agreement with each statement on a 7-point Likert scale from 1 (strongly disagree) to 7 (strongly agree).

During the third round, participants were emailed a link to an online questionnaire with the same statements alongside the median group rating for each item and a reminder of their own previous rating. Participants were instructed to re-rate their answers on the scale and change their response if they wanted. Participants were prompted to give their reasoning if their rating differed by more than one from the median [25]. Participants who had not completed the previous rounds were asked to rate each item on the 7-point scale without any information about the median group ratings.

### Analysis

Responses to the first questionnaire were interpreted using conventional content analysis [33]. Participant responses were read repeatedly to develop familiarity and immersion with the data [33]. Phrases that described key concepts were identified through systematic reading of the data [33]. Once concepts were identified they were compared to the wider dataset, similarly to the ‘reviewing themes’ stage of thematic analysis [34].

To adhere with the research aim of how to support autistic women during treatment for AN, concepts were configured into statements recommending ways of working (e.g. “Eating disorder staff should provide autistic women with more written information during treatment”) (similarly to Kelly, Jorm, Kitchener and Langlands [35]). Some themes did not contain clear recommendations and instead reflected knowledge participants felt staff would need (e.g. “Autistic women with anorexia might find weight gain distressing because of changes in their sensory experiences of their clothes or bodies”). The statements were created by looking at the original quotes representing each concept and using experts’ own wording where possible. The research team met regularly to discuss the content analysis and ensure the statements generated were relevant and did not contain unnecessary overlap.

Following round three, statements with a median of six or greater (labelled as “agree”) and an interquartile range (IQR) of less than or equal to one were considered to have reached consensus [25]. Statements were considered to have met a strong consensus if they had an IQR of less than one.

## Results

### Participant demographics

Table 2 shows the number of participants by expertise type who completed the third stage of the study. On average, the research experts (N = 7) had 7 years of professional experience (SD = 5.61 years; range 2–20), whereas clinical experts (N = 19) had 8.89 years of experience on average (SD = 8.75 years; range 1–30). Ten of the 20 EbE reported experience of service development or advocacy (M = 6.38 years; SD = 5.03; range 1–18). Other indices of experience for EbE, such as duration since diagnosis and recovery status, were not recorded. Data regarding the expertise types of participants at different points in the study are given in Fig. 1. Participants were living or working in the United Kingdom (n = 40), United States of America (n = 5), and Canada (n = 4).

**Table 2:** The number of participants by expertise type who completed the third round questionnaire

	Researcher	Clinician	Expert by experience	Total
Eating disorder	0	6	2	8
Autism	2	3	2	7
Both	5	10	16	31
Total	7	19	20	46

Experts were required to have expertise in eating disorders, autism, or both via experience as a researcher, clinician, or expert by experience

### Round 1

Ninety concepts were generated using content analysis and configured into statements. The statements were examined for overlap and 20 were removed in favour of more concrete statements about improving treatment. For example, the statement “Autistic women might be sensitive to particular noises, smells, or visual input, like lights around them” was removed in favour of “Buildings which are used for eating disorder treatment should be adapted to be more autism-friendly and less triggering to sensory sensitivities. This could be done by designing spaces together with autistic people.” In total 70 statements were retained for the second round questionnaire (see Additional file 1: Table S1).

### Round 2

Participants’ median agreement for each statement was calculated. The majority of statements had a high median agreement of six or more during round two (see Additional file 1: Table S1).

### Round 3

Of the original 70 statements, 56 reached consensus with 24 of these reaching strong consensus. For the medians and interquartile ranges (IQR) of all 70 statements, see Additional file 1: Table S1. The following section will discuss the 56 statements meeting consensus relating to the needs of autistic women, recommendations for eating disorders staff, and recommendations for services.

#### The needs of autistic women

Table 3 shows the statements meeting consensus regarding the needs of autistic women with AN and recommendations for treatment. Several recommendations focused on supporting women with managing aspects of the autistic experience, such as cognitive differences, sensory needs, and past social experiences. Some statements related to supporting autistic women to separate features of autism from ED difficulties, for example, by developing routines and exploring special interests that are unrelated to food.

**Table 3:** Statements reaching consensus (with a median of six or greater and an interquartile range of one or less) about the needs and recommendations for treatment of autistic women with anorexia nervosa

Statement	Interquartile range
*Autistic women with anorexia might*	
Feel they have to stick rigidly to meal plans given to them by services, which can be distressing when the meal plan can't be kept to	1
Be more likely to have other physical or mental health conditions which need considering during treatment	1
Need more time to complete and switch between tasks	1
Find changes in staff or unfamiliar staff more difficult to work with	0
Might be labelled as being ‘resistant’ due to not benefitting from traditional treatments	1
*Autistic women with anorexia might benefit from*	
Being supported to explore their special interests which are separate from food	1
Being treated by teams which have autistic staff members and therapists	0.75
Being treated by staff who are experienced in working with autistic people	0
Support and mentorship from other autistic people	1
*Eating disorder treatment for autistic women should*	
Involve support in a broader range of areas compared to non-autistic women with anorexia. Autistic women may benefit from support accessing education and employment, finding suitable accommodation, and finding support with daily tasks	1
Support autistic women to learn how to manage difficulties they might have with their attention, memory and organisational skills	1
Provide autistic women with information about the effects of starvation and refeeding on their bodies and minds	1
Support autistic women to understand what parts of their experiences are related to their autism and what parts are related to their eating disorder	1
Support autistic women with anorexia to develop new routines and rituals to replace unhelpful routines which are related to their eating disorder	1
Support autistic women to manage the demands of social relationships	1
Provide some autistic women with a trauma-informed approach or therapy to manage their past social experiences i.e. experiences of living in a society that can be negative about autism and autistic behaviour	1
Ensure that any specific psychological therapies like CBT are adapted for autistic women with anorexia	0.75
Support autistic women to explore their self-esteem and identity. This might involve exploring their identity as an autistic person	1
Support autistic women to identify what hunger cues feel like in their body	1
Adapt meal plans for autistic women to avoid their sensory sensitivities	0
Support autistic women to learn the best ways to regulate their sensory needs. This might involve using aids like weighted blankets, stim toys or headphones/ear plugs and allowing these to be used during mealtimes	1
Support autistic women to develop coping strategies that can be used in lots of different environments	1
Draw on the individual's strengths to help them recover	0
Include education and support for autistic women's families where possible	1

There were six statements meeting a strong consensus (with an IQR of less than one). These referenced that autistic women with AN may find changes in staff or new staff difficult and may benefit from being treated by teams experienced in working with autistic people, or with lived experience themselves. They suggested meal plans should avoid sensory sensitivities, psychological therapies should be adapted, and treatment should draw on autistic women’s strengths.

#### Recommendations for eating disorders staff

Table 4 shows the statements meeting consensus regarding how ED staff should work with autistic women and how they should be trained. The statements suggested staff can adjust their communication by asking specific questions, using more written communication, and using communication passports to document each individual’s communication needs.

**Table 4:** Statements reaching consensus (with a median of six or greater and an interquartile range of one or less) about how eating disorder staff should work with autistic women with anorexia nervosa and how they should be trained

Statement	Interquartile range
*Eating disorder staff might*	
Have concerns that non-autistic patients will think it is unfair that autistic women with anorexia receive different treatment	1
Find it difficult to work with autistic women with anorexia because of pressure on services and a lack of time	1
*Eating disorder staff should*	
Change how they communicate with autistic women. Staff should try and ask specific questions instead of open ones. Staff should try and be clear about what they mean and check they have been understood	1
Develop 'communication passports' with autistic women so that all staff know the individuals' unique communication needs	1
Be aware that some autistic women benefit from staff communicating with them in a way that puts less pressure on them. This might involve asking them to do things in a less directive or demanding way	1
Provide autistic women with more written information during treatment	1
Be accepting of autistic women's need to stim (make repetitive actions/movement in order to get regulating sensory input)	0
Develop trusting and empathetic therapeutic relationships with autistic women with anorexia nervosa	0
Be aware that it might take more time to develop therapeutic relationships with autistic women with anorexia compared to with non-autistic women with anorexia	0.75
Be aware that autistic women with anorexia may be more likely to feel blamed by staff and should try and communicate information in a sensitive, non-blaming way	1
*A training package for eating disorder staff should*	
Be developed and delivered together with autistic women	0
Be delivered to all eating disorder staff so that they have a better understanding of autism	0
Teach staff about how autism might look different in women compared to men	0.25
Draw attention to autistic people's strengths and theories of neurodiversity instead of just deficit-based information	0
Ensure staff are able to screen patients for autism	1
Ensure staff are able to distinguish between anorexia and ARFID (avoidant/restrictive food intake disorder, where a person finds eating particular foods very aversive due to things like texture and taste)	0.75
Teach staff about the ways that anorexia and autism may interact and affect each other	0
Ensure staff are able to distinguish between eating disorder behaviour and autistic behaviour, and train them to avoid setting treatment goals which aim to change autistic behaviour	0
Teach staff about meltdowns and shutdowns that autistic women may experience when overwhelmed, and how to avoid and respond to these	0
Teach staff how to adapt interventions so they are more suitable for autistic women	0

Several statements reached strong consensus and all but one of the statements relating to staff training met strong consensus. Participants recommended a training package be developed and delivered together with autistic women and provided to all staff. Training was recommended to cover a range of topics including information about: autistic strengths; autistic meltdowns and shutdowns; how to adapt interventions; how autism and AN interact; and the importance of treatment goals not modifying autistic behaviours. The statements referred to the importance of building trusting relationships with autistic women and highlighted that this may take longer compared to non-autistic women.

#### Recommendations for services

Table 5 shows the statements meeting consensus that make recommendations about service-level changes to support autistic women with AN. Recommendations included adapting buildings to be less triggering to sensory sensitivities and developing online information specifically aimed at autistic women with AN.

**Table 5:** Statements reaching consensus (with a median of six or greater and an interquartile range of one or less) about how services can support autistic women with anorexia nervosa

Statement	Interquartile range
*Eating disorder services should*	
Support autistic women with anorexia to manage changes in treatment. This might involve having more warning about changes or having longer transitions between teams	0
Ensure that any rules (including ‘unwritten’ rules) are fully explained and justified, as autistic women may find it difficult to adjust to different rules in different services	0.75
Provide autistic women with anorexia with structured, clear treatment plans with goals which are broken into achievable steps	0.75
Work collaboratively with autistic women to tailor treatment to their individual needs, as what is helpful for one autistic woman may be unhelpful for another	0
Ensure different staff to have a consistent approach with autistic women with anorexia	1
Ensure staff working with autistic women with anorexia need regular supervision to discuss their work	1
Adapt buildings to be more autism-friendly and less triggering to sensory sensitivities. This could be done by designing spaces together with autistic people	1
Be adapted to be more accessible for autistic people, i.e. communicating via text messages and emails as well as phone calls	0
Provide more online information aimed at autistic women with anorexia i.e. educational information, blog posts, community spaces	1
Use the PEACE pathway (Pathway for Eating disorders and Autism developed from Clinical Experience, developed by Kings College London and South London and Maudsley NHS Trust)	0.75
Be aware that traditional treatment and environments which are aimed at neurotypical people can be harmful to autistic women with anorexia	1
Be aware that some changes to treatment that would be helpful for autistic women might also be helpful for non-autistic women	1

The statements meeting strong consensus suggested autistic women would benefit from services that: supported adjustment to changes in treatment; explained rules clearly; provided clear treatment plans that were broken into steps; provided a range of communication options; and worked collaboratively with autistic women to tailor treatment to individual needs. There was also strong consensus for using the PEACE pathway.

Across all statement types, some similarities were identified. Many statements suggested staff should attempt to differentiate between behaviours relating to autism and those relating to AN. This was reflected by statements with strong consensus that recommended staff receive training on the interaction between autism and AN (IQR = 0), and that they set treatment goals which do not aim to change autistic behaviours (IQR = 0). Statements recommended supporting women to cope with autistic traits that might affect their ED. This included: coping with cognitive difficulties (IQR = 1); regulating sensory needs (IQR = 1); developing new routines (IQR = 1) and exploring special interests unrelated to the ED (IQR = 1).

Many statements referred to the need for staff, services, and treatment to accommodate autistic traits. Examples included the suggestion that meal plans should avoid sensory sensitivities (IQR = 0), staff should accept autistic women’s need to ‘stim’ (IQR = 0), and services should provide more warning about changes (IQR = 0) and ensure treatment plans are clear and structured (IQR = 0.75), and communication with autistic people requires adaptation (IQR = 1).

Finally, many statements emphasised the importance of embedding the autistic voice within training and treatment. This was reflected in statements that recommended that autistic women should be included in the development and delivery of training (IQR = 0), that staff teams should include autistic staff members (IQR = 0.75), and that services should work with autistic women to tailor treatment to their individual needs (IQR = 0).

## Discussion

Using the Delphi method, the current study systematically explored the views of researchers, clinicians and EbE on how to best support autistic women with AN receiving treatment for an ED. Based on the responses of participants, 70 statements were generated and following three Delphi rounds, 56 statements reached consensus. Participants recommended that treatment distinguish between autism- and AN-related behaviours, that autistic traits should be accommodated, and that autistic people should be included in the development and delivery of care.

### Distinguishing between autism and anorexia nervosa

Experts in the current study agreed it was important for staff to be trained to distinguish between autism- and AN-related behaviours to avoid setting targets which aim to change autism-related behaviours. Additionally, experts suggested it is useful for autistic women to be supported to understand which of their experiences are related to autism and which are related to an ED. Brede et al. [13] autism-specific model of restrictive eating provides a potential framework for distinguishing between autism- and AN-related behaviours. Within the model, autism-related traits can lead to restricted eating through a direct pathway e.g., sensory sensitivities leading to the avoidance of certain foods. In addition, autistic traits can precipitate restrictive eating indirectly. For example, an autistic person might struggle with uncertainty and experience anxiety as a result. Restricted eating might develop to reduce anxiety, providing a sense of control and predictability [13]. Notably, the same autistic trait may lead to restricted eating via both direct and indirect pathways [13]. This complex interplay between autism and AN may make it challenging for staff to identify autistic precursors to AN.

Some statements meeting consensus suggested autistic women with AN might benefit from treatment targeting a different range of difficulties compared to non-autistic women. Participants suggested autistic women might benefit from support with daily tasks, managing difficulties with attention and organisational skills, regulating their sensory needs, and managing the impact of traumatic social experiences. Without support for these differences, autistic women might partly cope with them through restricting eating [13]. Some statements suggested autistic women would benefit from support to develop new routines and explore special interests that are unrelated to their ED. AN-related routines and special interests are an example of a direct effect of autistic traits on ED symptoms [13].

The treatment targets proposed in this study contrast with traditional treatments such as Enhanced Cognitive Behaviour Therapy (CBT-E) [36] and the Maudsley Model of Anorexia Nervosa Treatment for Adults (MANTRA) [37]. Traditional ED treatments have focused on addressing overevaluation of weight and shape, pro-anorectic beliefs, perfectionism, cognitive inflexibility, low self-esteem, difficulty coping with emotions, and difficulties with relationships [36–41]. Many of these factors are theorised to result in part from the effects of starvation, meaning weight restoration is one of the first aims in CBT-E for individuals with AN [42]. Indeed, there is evidence that traits such as weak central coherence are mainly evident in a starvation state in these individuals [40]. CBT-E also targets some of these traits using cognitive and behavioural methods, e.g., perfectionism. However, Brede et al. [13] model highlights features of autism that facilitate the pathway into and maintenance of an ED. Clinicians should be aware that the features of EDs in autistic individuals may have different causes that precede the ED and may therefore be less amenable to change via weight restoration and traditional cognitive-behavioural strategies. Cognitive difficulties might be an autistic feature [43] that precipitates or maintains EDs for autistic women [13]. As a result, learning to manage and understand cognitive differences may be an important part of AN treatment for autistic women.

Despite the importance of early intervention in ED treatment for non-autistic people [44, 45], none of the statements reaching consensus in the present study explicitly referred to early intervention. As many women with EDs do not receive an autism diagnosis until after they have had contact with ED services [15], it may be that early intervention for ED symptoms was less salient to participants when considering the needs of this client group. As the present study suggests that autistic women with AN may benefit from support that targets a wider range of difficulties which might precipitate their ED, it is useful to consider whether this may be useful as part of early intervention for autistic women who may be developing an ED. The role of and adaptations to early intervention for autistic women with AN is an important focus for future research.

### Accommodating autistic traits

Many statements reaching consensus recommended that women’s autistic traits should be accommodated during ED treatment. This was discussed in relation to adapting treatment to avoid sensory sensitivities, adapting communication, and being flexible to the needs of individuals.

Experts in the current study agreed that meal plans and treatment environments should be adapted to avoid autistic women’s sensory sensitivities. This is consistent with previous research where autistic people reported that sensory inputs such as certain lights, colours, noises, and disliked food tastes and textures trigger anxiety [46]. Previous research has suggested autistic women with AN might restrict food intake in response to sensory sensitivities [13], whilst sensory sensitivities have also been associated more broadly with ED behaviours in autistic adults [47]. At the level of the treatment environment, it has previously been recognised by autistic women, parents and clinicians that accommodating sensory sensitivities withing the ED service environment would be beneficial for autistic women [15].

Standard ED treatments such as CBT-E typically formulate the avoidance of foods as relating to over-evaluation of weight and shape and recommend reintroducing ‘feared foods’ [38, 39]. However, an approach that accommodates autistic traits would recognise that sensory sensitivities may be the driver of food avoidance in many autistic people with AN.

Individual differences are relevant when considering adapting menus to avoid sensory sensitivities. The PEACE pathway [18, 19] provides an additional menu, which is designed to accommodate common sensory sensitivities by utilising bland, low odour, homogenous textured foods. However, this may not encompass the diversity of sensory differences experienced by autistic people, including hyposensitivity and seeking food with more intense sensory qualities [46, 48]. Recommendations in the current study highlight the need to adapt treatment to the individual autistic person.

Several statements met consensus suggesting staff should adapt their communication in a variety of ways when working with autistic women, including asking specific questions, providing more written information, and creating individual ‘passports’ to detail communication preferences. These findings are consistent with the opinions of autistic women being treated for AN who highly valued healthcare professionals who adapted their communication to meet their needs [15]. The data also reflects recognition in the literature of different communication preferences (e.g. face-to-face vs. written) among autistic people [49]. Communication passports are used for autistic adults in inpatient psychiatric services [50], suggesting they could be usefully adapted autistic women with AN.

### Embedding the autistic voice within training and treatment

Experts in the current study agreed that autistic women should be involved with the development and delivery of training for staff. This aligns with wider discussion of the importance of including stakeholders in the design of mental health services and systems [51], along with recognition that meaningful co-production can be challenging [52]. Training in co-production (a ‘co-production curriculum’) may support success [53]. However, co-production with autistic people also needs to consider their specific needs, including ways to support different communication preferences and consideration of necessary environmental adaptations [54].

Experts in the current study also agreed that services should work with autistic women to ensure treatment is tailored to their individual needs, avoiding a ‘one size fits all’ approach. Autistic people accessing mental health services felt support is not tailored to their needs, particularly individual differences in autism, which can result in withdrawal from services [55]. Similarly, they identified that individually tailored care was important in enabling them to access mental health services [55, 56]. To ensure treatment is tailored to the needs of autistic women, it is important to consider how individual autistic women define recovery from an ED and how this may or may not differ from the definitions of non-autistic people [57].

### Clinical implications

The current study has implications for existing guidelines for adapting care for autistic women with AN and could be utilised to develop a training package for staff. Recommendations shared between the current study and the PEACE pathway include training staff to screen for autism and making adaptations to the physical environment [18, 19]. Statements from the current study recommend that a training package be developed and delivered in partnership with autistic women with AN. Training should enable staff to distinguish between autism- and AN-related behaviours. Staff should be trained to support women to cope with autism-related difficulties that indirectly affect their AN, such as difficulties with attention and past and present social experiences. However, staff should also be trained to avoid treatment targets that focus on reducing autistic behaviours. This approach reflects that autism is a lifelong neurodevelopmental condition that requires support and understanding, and which is characterised by strengths as well as difference and difficulties [58]. This is a different frame to the drive for treatment of AN, which is a serious psychiatric condition with high mortality [4]. Reflecting the consensus statements of our participants, training staff in neurodiversity may support understanding of this critical distinction.

### Strengths and limitations

This research represents a systematic investigation of how to support autistic women during treatment for AN, with transparent contributions from professionals and EbE. This is the first time this group’s needs have been investigated in this way and we present consensus recommendations for services and professionals.

The current study included experts from across the UK, USA, and Canada, enabling wider generalisation than a country-specific project. However, it is unclear to what extent the recommendations would be endorsed in other countries, particularly those where understanding of autism is less progressed [59]. As Delphi studies involve storing data across multiple rounds, the researchers collected minimal data about each expert (years of experience) to preserve participants’ anonymity. This precluded more fine-grained analyses. For example, we were unable to compare recommendations by professional background, or consider if there was any impact on responses depending on whether the EbE was a person with or recovered from an ED, or were a family member or carer.

The present research investigated how to support autistic women with AN. Future research should investigate whether these recommendations are useful for autistic people with other gender identities, including men and people whose gender identity that does not align with sex. This is particularly relevant given the overrepresentation of gender-diverse and transgender people within the autistic community [60–62]. Additionally, there has been a historical lack of research into ED in men [63, 64] and research suggests that autistic men may also experience higher levels of non-restrictive ED symptoms [65]. Similarly, by focussing on women with AN, our data do not extend to other types of ED although we predict many of the recommendations would be applicable.

There was attrition of experts between the initial recruitment phase and the first-round questionnaire, which is a common difficulty in Delphi studies [27]. Particularly, the number of clinicians and researchers in the first round was notably smaller than the number of EbE. Additional experts were recruited in the third round to ensure a more equal number of clinicians and EbE. There remained fewer researchers compared to clinicians and EbE in the current sample. A potential limitation of this is that there could be a risk that the results of this study are less reflective of the existing empirical literature. However, the results of the present study appear consistent with the existing literature on autism and AN. All participants self-identified as experts in the areas of autism, ED, or both, which was necessary for the integrity of the Delphi method (Okoli & Pawlowski [28]). However, it is unclear if the wider community of stakeholders would agree with the study conclusions. Future research should explore the wider acceptability of these recommendations and the impact of their implementation on outcomes for autistic women with AN.

## Conclusion

We used the Delphi method to systematically investigate the views of professionals and EbE on the best ways to support autistic women in treatment for an AN. Key themes emerging from our recommendations were: treatment should differentiate between autism- and AN-related difficulties; autistic traits should be accommodated and supported rather than ‘treated’, and the autistic voice should be present in both the development and delivery of care. Ultimately, the recommendations advocate for training that enhances the understanding of autistic women with AN within ED services. Future research should seek to establish the effectiveness of these training recommendations in producing positive change for autistic women with AN.

## Supplementary Information

**Additional file 1**: **Table S1**. Statements generated in the content analysis, median agreement with each statement in round 2, and median agreement and interquartile range for each statement in round 3. Statements which reached consensus are marked with an asterisk.

## Data Availability

The datasets generated and/or analysed during the current study are not publicly available due to UK General Data Protection Regulation (UK GDPR) but are available from the corresponding author on reasonable request.

## Abbreviations

AN: Anorexia nervosa
ARFID: Avoidant/restrictive food intake disorder
CBT-E: Enhanced cognitive behaviour therapy
EbE: Expert(s) by lived experience
ED: Eating disorder
IQR: Interquartile range
MANTRA: Maudsley model of anorexia nervosa treatment for adults
PEACE Pathway: Pathway for eating disorders and autism developed from clinical experience
