# Supplementary material for: “Work WITH us”: a Delphi study about improving eating disorder treatment for autistic women with anorexia nervosa
Source: J Eat Disord. 2023 Feb 9;11:17. doi: 10.1186/s40337-023-00740-z (PMC9909870; doi:10.1186/s40337-023-00740-z)
Supplement: Supplementary file 1 — Additional file 1: Table S1. Statements generated in the content analysis, median agreement with each statement in round 2, and median agreement and interquartile range for each statement in round 3. Statements which reached consensus are marked with an asterisk. [file 40337_2023_740_MOESM1_ESM.docx]

**Table S1**

*Statements generated in the content analysis, median agreement with each statement in round 2, and median agreement and interquartile range for each statement in round 3. Statements which reached consensus are marked with an asterisk.*

| **Statement** | **Round 2 Median agreement** | **Round 3 Median agreement** | **Round 3 Interquartile range** |
| --- | --- | --- | --- |
| **Autistic women with anorexia might...** |  |  |  |
| ...feel they have to stick rigidly to meal plans given to them by services, which can be distressing when the meal plan can't be kept to. | 6.5 | 6 | 1* |
| ...be more likely to have other physical or mental health conditions which need considering during treatment | 7 | 7 | 1* |
| ...be more likely to self-harm than non-autistic women with anorexia | 5 | 4.5 | 2 |
| ...find weight gain distressing because of changes in their sensory experiences of their clothes or bodies | 6 | 6 | 2 |
| ...need more time to complete and switch between tasks. | 6.5 | 7 | 1* |
| ...find changes in staff or unfamiliar staff more difficult to work with | 7 | 7 | 0* |
| ...not benefit from group therapy | 5.5 | 5 | 2 |
| ...might be labelled as being ‘resistant’ due to not benefitting from traditional treatments | 7 | 7 | 1* |
| ...have a different response to mental health medication compared to non-autistic people | 6 | 5 | 1.75 |
| **Autistic women with anorexia might benefit from...** |  |  |  |
| ...being supported to explore their special interests which are separate from food. | 7 | 7 | 1* |
| ...being treated by teams which have autistic staff members and therapists. | 7 | 7 | 0.75* |
| ...being treated by staff who are experienced in working with autistic people | 7 | 7 | 0* |
| ...support and mentorship from other autistic people | 7 | 7 | 1* |
| ...receiving treatment as an inpatient instead of receiving treatment in the community | 4 | 3 | 2 |

| **Statement** | **Round 2 Median agreement** | **Round 3 Median agreement** | **Round 3 Interquartile range** |
| --- | --- | --- | --- |
| **Eating disorder staff might...** |  |  |  |
| ...have negative beliefs and reactions towards autistic behaviour e.g. believing that meltdowns are 'tantrums' or that lack of eye contact is impolite | 7 | 6.5 | 2 |
| ...have concerns that non-autistic patients will think it is unfair that autistic women with anorexia receive different treatment | 6 | 6 | 1* |
| ...find it harder to relate to an autistic woman's experiences and understand her communication style compared to when they work with non-autistic women | 6 | 6 | 2 |
| ...find it difficult to work with autistic women with anorexia because of pressure on services and a lack of time | 6 | 6 | 1* |
| **Eating disorder staff should...** |  |  |  |
| ...change how they communicate with autistic women. Staff should try and ask specific questions instead of open ones. Staff should try and be clear about what they mean and check they have been understood. | 7 | 7 | 1* |
| ...develop 'communication passports' with autistic women so that all staff know the individuals' unique communication needs | 7 | 7 | 1* |
| ...be aware that some autistic women benefit from staff communicating with them in a way that puts less pressure on them. This might involve asking them to do things in a less directive or demanding way. | 6.5 | 7 | 1* |
| ...provide autistic women with more written information during treatment | 7 | 7 | 1* |
| ...be accepting of autistic women's need to stim (make repetitive actions/movement in order to get regulating sensory input) | 7 | 7 | 0* |
| ...develop trusting and empathetic therapeutic relationships with autistic women with anorexia nervosa. | 7 | 7 | 0* |
| ...be aware that it might take more time to develop therapeutic relationships with autistic women with anorexia compared to with non-autistic women with anorexia. | 7 | 7 | 0.75* |
| ...be aware that autistic women with anorexia may be more likely to feel blamed by staff and should try and communicate information in a sensitive, non-blaming way. | 7 | 7 | 1* |
| **Eating disorder treatment for autistic women should...** |  |  |  |
| ...usually place less focus on body image than treatment for non-autistic women | 5 | 5 | 1.75 |
| ...be aware that autistic people are more likely to be gender non-conforming, non-binary or transgender. This can have an impact on their body image and might need to be explored during treatment. | 6 | 6 | 1.75 |
| ...involve asking autistic people with anorexia screening questions about their gender identity. | 6 | 6 | 2 |
| ...involve support in a broader range of areas compared to non-autistic women with anorexia. Autistic women may benefit from support accessing education and employment, finding suitable accommodation, and finding support with daily tasks | 6.5 | 6 | 1* |
| ...support autistic women to learn how to manage difficulties they might have with their attention, memory and organisational skills | 6 | 6 | 1* |
| ...provide autistic women with information about the effects of starvation and refeeding on their bodies and minds | 6.5 | 7 | 1* |
| **Statement** | **Round 2 Median agreement** | **Round 3 Median agreement** | **Round 3 Interquartile range** |
| **Eating disorder treatment for autistic women should...** |  |  |  |
| ...support autistic women to understand what parts of their experiences are related to their autism and what parts are related to their eating disorder | 7 | 7 | 1* |
| ...support autistic women with anorexia to develop new routines and rituals to replace unhelpful routines which are related to their eating disorder | 7 | 7 | 1* |
| ...support autistic women to develop social skills | 5.5 | 5 | 2 |
| ...support autistic women to manage the demands of social relationships | 6 | 6 | 1* |
| ...provide some autistic women with a trauma-informed approach or therapy to manage their past social experiences i.e. experiences of living in a society that can be negative about autism and autistic behaviour | 7 | 7 | 1* |
| ...ensure that any specific psychological therapies like CBT are adapted for autistic women with anorexia. | 7 | 7 | 0.75* |
| ...involve a longer course of psychological therapy. | 6 | 6 | 2 |
| ...include specific therapy aimed at identifying and regulating emotions | 6 | 6 | 2 |
| ...support autistic women to explore their self-esteem and identity. This might involve exploring their identity as an autistic person. | 6 | 6 | 1* |
| ...support autistic women to identify what hunger cues feel like in their body | 7 | 7 | 1* |
| ...adapt meal plans for autistic women to avoid their sensory sensitivities. | 7 | 7 | 0* |
| ...encourage autistic women with anorexia to try new foods and new food textures | 4 | 4 | 2 |
| ...support autistic women to learn the best ways to regulate their sensory needs. This might involve using aids like weighted blankets, stim toys or headphones/ear plugs and allowing these to be used during mealtimes. | 7 | 7 | 1* |
| ...support autistic women to develop coping strategies that can be used in lots of different environments | 7 | 7 | 1* |
| ...draw on the individual's strengths to help them recover | 7 | 7 | 0* |
| ...include education and support for autistic women's families where possible. | 6 | 6 | 1* |
| **A training package for eating disorder staff should...** |  |  |  |
| ...be developed and delivered together with autistic women | 7 | 7 | 0* |
| ...be delivered to all eating disorder staff so that they have a better understanding of autism | 7 | 7 | 0* |
| ...teach staff about how autism might look different in women compared to men | 7 | 7 | 0.25* |
| ...draw attention to autistic people's strengths and theories of neurodiversity instead of just deficit-based information. | 7 | 7 | 0* |
| **tatement** | **Round 2 Median agreement** | **Round 3 Median agreement** | **Round 3 Interquartile range** |
| **A training package for eating disorder staff should...** |  |  |  |
| ...ensure staff are able to screen patients for autism | 6.5 | 7 | 1* |
| ...ensure staff are able to distinguish between anorexia and ARFID (avoidant/restrictive food intake disorder, where a person finds eating particular foods very aversive due to things like texture and taste). | 7 | 7 | 0.75* |
| ...teach staff about the ways that anorexia and autism may interact and affect each other | 7 | 7 | 0* |
| ...ensure staff are able to distinguish between eating disorder behaviour and autistic behaviour, and train them to avoid setting treatment goals which aim to change autistic behaviour | 7 | 7 | 0* |
| ...teach staff about meltdowns and shutdowns that autistic women may experience when overwhelmed, and how to avoid and respond to these | 7 | 7 | 0* |
| ...teach staff how to adapt interventions so they are more suitable for autistic women | 7 | 7 | 0* |
| **Eating disorder services should...** |  |  |  |
| ...support autistic women with anorexia to manage changes in treatment. This might involve having more warning about changes or having longer transitions between teams. | 7 | 7 | 0* |
| ...ensure that any rules (including ‘unwritten’ rules) are fully explained and justified, as autistic women may find it difficult to adjust to different rules in different services. | 7 | 7 | 0.75* |
| ...provide autistic women with anorexia with structured, clear treatment plans with goals which are broken into achievable steps. | 7 | 7 | 0.75* |
| ...work collaboratively with autistic women to tailor treatment to their individual needs, as what is helpful for one autistic woman may be unhelpful for another. | 7 | 7 | 0* |
| ...ensure different staff to have a consistent approach with autistic women with anorexia | 6 | 6 | 1* |
| ...ensure staff working with autistic women with anorexia need regular supervision to discuss their work. | 6 | 7 | 1* |
| ...adapt buildings to be more autism-friendly and less triggering to sensory sensitivities. This could be done by designing spaces together with autistic people. | 6.5 | 7 | 1* |
| ...be adapted to be more accessible for autistic people, i.e. communicating via text messages and emails as well as phone calls | 7 | 7 | 0* |
| ...provide more online information aimed at autistic women with anorexia i.e. educational information, blog posts, community spaces | 6.5 | 6 | 1* |
| ...use the PEACE pathway (Pathway for Eating disorders and Autism developed from Clinical Experience, developed by Kings College London and South London and Maudsley NHS Trust). | 6 | 6 | 0.75* |
| ..be aware that traditional treatment and environments which are aimed at neurotypical people can be harmful to autistic women with anorexia | 7 | 7 | 1* |
| ...be aware that some changes to treatment that would be helpful for autistic women might also be helpful for non-autistic women. | 7 | 7 | 1* |
